# Elevated alcohol consumption following alcohol cue exposure is partially mediated by reduced inhibitory control and increased craving

**DOI:** 10.1007/s00213-017-4694-6

**Published:** 2017-07-25

**Authors:** Matt Field, Andrew Jones

**Affiliations:** 1 Department of Psychological Sciences, University of Liverpool, Liverpool, L69 7ZA, UK; 2 UK Centre for Tobacco and Alcohol Studies, Liverpool, UK

**Keywords:** Alcohol, Craving, Cue exposure, Inhibitory control, Stop-signal task

## Abstract

**Rationale:**

Exposure to alcohol-related cues leads to increased alcohol consumption, and this may be partially attributable to momentarily impaired impulse control.

**Objectives:**

We investigated if exposure to alcohol cues would impair inhibitory control and if the extent of this impairment would partially mediate the effect of alcohol cues on subsequent voluntary alcohol consumption.

**Methods:**

We recruited 81 heavy drinkers (50 female) who completed baseline measures of inhibitory control (stop-signal task) and subjective craving before random allocation to an alcohol cue exposure or control group. The alcohol cue exposure group then completed a second stop-signal task (with embedded alcohol cues) with concurrent exposure to olfactory alcohol cues, in an alcohol context. The control group completed a second stop-signal task (with embedded water cues), accompanied by exposure to water cues, in a neutral context. Then, subjective craving and ad libitum alcohol consumption were measured in all participants.

**Results:**

Inhibitory control worsened (compared to baseline) to a greater extent in the alcohol cue exposure group compared to the control group. Craving and ad libitum alcohol consumption were elevated in the alcohol cue exposure group compared to the control group, although the group difference in alcohol consumption fell short of statistical significance. In support of our hypotheses, multiple mediation analyses demonstrated that elevated ad libitum alcohol consumption following alcohol cue exposure was partially mediated by both impaired inhibitory control and increased craving.

**Conclusions:**

These findings suggest that state fluctuations in inhibitory control are a potential mechanism through which alcohol cues increase drinking behaviour.

**Electronic supplementary material:**

The online version of this article (doi:10.1007/s00213-017-4694-6) contains supplementary material, which is available to authorized users.

## Introduction

In the substance use literature, ‘cue reactivity’ refers to the observation that exposure to substance-related cues (such as the sight or smell of alcoholic beverages) evokes elevations in subjective craving and physiological arousal and increases the likelihood of substance use (Carter and Tiffany 1999; Veilleux and Skinner 2015). According to a number of theories, the associative learning mechanisms that underlie cue reactivity play a critical role in the development and maintenance of addiction (substance use disorders) and in the relapse to substance use after periods of abstinence (Goldstein and Volkow 2002; Robinson and Berridge 1993; Stacy and Wiers 2010). These claims are supported by findings from studies that used ecological momentary assessment (EMA) methods, which confirmed the influence of substance-related cues on craving and substance use in naturalistic settings, outside of the laboratory (Fatseas et al. 2015; Serre et al. 2015).

There is broad agreement that associative learning mechanisms underlie the development of these responses to substance-related cues. However, there is ambiguity about the psychological mechanisms that explain the influence of substance-related cues on substance-seeking behaviour and overt consumption. Multiple processes are likely to be involved, including the Pavlovian-to-instrumental transfer (PIT; see Hogarth et al. 2014), habitual stimulus-response associations (Tiffany 1990) and activation of automatic appetitive motivational processes (e.g. automatic approach and attentional biases) that prompt substance use irrespective of intentions to consume (or intentions to refrain from consumption) (Stacy and Wiers 2010). There is evidence in support of each of these accounts (Miller and Gold 1994; Stacy and Wiers 2010; Hogarth and Chase 2012); however, none of these processes in isolation is likely to explain all of the variance in the effects of cues on behaviour, and it is likely that additional psychological processes are involved. In the present article, we investigated if impairments in inhibitory control that arise during and after exposure to alcohol-related cues might also partially account for the influence of those cues on subsequent alcohol consumption.

Inhibitory control refers to the ability to effectively stop, change or delay behaviour (Logan et al. 1984), and it is a component of broader constructs such as impulsivity, executive dysfunction and self-control, each of which has been implicated in addiction (Baler and Volkow 2006; Bickel et al. 2012; Fujita 2011). Inhibitory control can be measured objectively using computerised tasks such as the stop-signal or go/no-go tasks (Diamond 2013), both of which require individuals to inhibit motor behaviour in response to a cue or signal to inhibit. Deficient inhibitory control plays an important and potentially causal role in alcohol and other substance use disorders. Deficits in inhibitory control have been observed in alcohol-dependent patients, compared to healthy controls (Smith et al. 2014). Furthermore, within non-dependent alcohol consumers, inhibitory control is worse in those who drink more heavily (Christiansen et al. 2012b; Houston et al. 2014; Smith et al. 2014). Longitudinal studies have demonstrated that inhibitory control predicts progression from heavy drinking to dependence (Rubio et al. 2008) and the likelihood of relapse following treatment (Rupp et al. 2016). Furthermore, the development of inhibitory control during childhood and adolescence is closely linked to the initiation and escalation of substance use, including alcohol consumption (Fernie et al. 2013; Nigg et al. 2006). It is also likely that the causal relationship between heavy drinking and impaired inhibitory control is bidirectional, because chronic heavy drinking may result in brain damage that results in impaired inhibitory control (Lopez-Caneda et al. 2014).

Inhibitory control may also moderate individual differences in cue reactivity: Heavy drinkers with impaired inhibitory control report enhanced craving after exposure to alcohol-related cues (Papachristou et al. 2013; Papachristou et al. 2012). Despite the presence of these between-group differences, laboratory research suggests that within alcohol consumers, inhibitory control is not a stable trait. Rather, it appears to fluctuate in response to internal (e.g. self-control depletion, Huizenga et al. 2012; arousal, Jones and Field 2015; Verbruggen and De Houwer 2007) and environmental events such as exposure to alcohol-related cues (Czapla et al. 2015; Gauggel et al. 2010; Jones and Field 2015; Monk et al. 2016; Petit et al. 2012; Weafer and Fillmore 2012a, 2014; Zack et al. 2011). These momentary ‘state’ fluctuations in inhibitory control during exposure to alcohol-related cues may increase the likelihood that people will drink alcohol (or increase the amount that they will consume) because they are unable to effectively regulate behaviour in the face of temptation (de Wit 2009; Jones et al. 2013a).

In a previous study (Jones et al. 2013b), we attempted to directly test the hypothesis that a deficit in inhibitory control was a mechanism through which exposure to alcohol-related cues prompted increases in alcohol consumption. In this study, non-dependent heavy drinkers were exposed to olfactory alcohol (or water) cues before they completed a stop-signal task followed by a bogus ‘taste test’ which permitted the measurement of the amount of beer that they would voluntarily consume (see Jones et al. 2016a). Compared to a group of participants who had been exposed to control (water) cues, participants who had been exposed to alcohol cues reported elevated craving and consumed more beer during the taste test. However, we did not observe the predicted impairment in inhibitory control in participants who had been exposed to alcohol cues. A potential methodological issue with this study was that alcohol-related cues were presented *before* but not *during* the requirement to inhibit, unlike in many previous studies (Jones and Field 2015; Weafer and Fillmore 2012a, 2014), and therefore, any effect of alcohol cues on inhibitory control may have dissipated when inhibitory control was actually measured. A further limitation is that we only measured inhibitory control in each participant once, after they had been exposed to alcohol cues; therefore, we were unable to examine how inhibitory control changed *within* individuals following alcohol cue exposure.

The aim of the current study was to extend our earlier study by investigating whether exposure to visual, olfactory and contextual alcohol-related cues would lead to a transient *within-subject* impairment in inhibitory control in a sample of heavy drinkers and whether this would be associated with subsequent alcohol consumption. We used a mixed experimental design in which participants were exposed to either alcohol-related cues or control (water-related cues), and their inhibitory control was measured immediately before and then during cue exposure. We had three primary hypotheses: (i) Inhibitory control would be impaired during exposure to alcohol-related cues compared to during exposure to control (water-related) cues; (ii) participants who had been exposed to alcohol-related cues would consume more alcohol during a bogus taste test than participants who had been exposed to water-related cues; and (iii) the hypothesised group difference in alcohol consumption after cue exposure would be partially mediated by the change in inhibitory control. We also measured changes in craving in an attempt to replicate previous demonstrations that alcohol cues would increase craving (Carter and Tiffany 1999; Veilleux and Skinner 2015) and in order to investigate if elevated craving after cue exposure would also partially mediate the effect of alcohol cues on alcohol consumption.

## Method

### Participants

We recruited 81 participants (50 female; mean age 19.99 ± 3.05). The target sample size of *N* = 80 was based on an a priori power calculation for identifying the effect of alcohol cue exposure on ad libitum alcohol consumption (*d* = 0.82, based on Jones et al. 2013b) with 95% power and *α* = 0.05. We recruited an additional participant because one participant had missing data on self-report scales (see below). We allocated participants to groups using a random number generator to ensure unbiased randomisation (Suresh 2011) which led to slightly unequal group sizes (N2/N1 ratio = 0.84); however, this did not change the outcome of our power calculation. Participants were recruited from the student and staff population at the University of Liverpool, using advertisements placed around the campus. Participants were required to be aged over 18 years, report liking beer and drink in excess of the UK government guidelines for sensible drinking (at the time, these were 14 units per week for females and 21 units for males, with 1 unit = 8 g alcohol). Note that the guidelines for males were revised downwards to 14 units in January 2016 after the recruitment for this study was complete. Individuals were excluded from participation if they had a current or previous diagnosis of alcohol or other substance use disorder or attention deficit hyperactivity disorder, which was assessed via self-report during email screening. The study was approved by the University of Liverpool Research Ethics committee.

### Materials

#### Baseline stop-signal task (based on Verbruggen and Logan 2008)

The beginning of each trial was signalled by a fixation cross (‘+’) that was presented in the centre of the screen for 500 ms. This was immediately followed by a go stimulus—an arrow that pointed left or right—for 1000 ms. Participants were instructed to make a speeded response to the direction of the arrow, by pressing one of two labelled keys on the keyboard. Go stimuli were uninterrupted on 75% of trials (go trials). The remaining 25% of the trials were stop trials: An auditory tone (the stop signal) was presented at a variable delay after the presentation of the go stimulus. Participants were instructed to inhibit their categorisation response whenever they heard the stop signal. The delay between the go stimulus and stop signal onset (stop-signal delay (SSD)) was adjusted using a dynamic tracking procedure. The initial SSD was 250 ms. If the participants successfully inhibited their response, the SSD increased by 50 ms on the subsequent stop trial (making inhibition more difficult), whereas if the participant failed to inhibit, the SSD decreased by 50 ms on the subsequent stop trial (making inhibition easier).

Participants completed 16 practice trials, before 3 blocks of 64 trials. In each block, there were 48 go trials and 16 stop trials. Trial order was randomised for each participant, and the task took approximately 10 min to complete.

#### Cued stop-signal tasks with in vivo cue exposure

During these tasks, participants made speeded responses to images of beverages that were presented on the screen. Participants were instructed to quickly categorise whether the beverage depicted was contained in a bottle or a glass, by pressing one of two labelled keys on the keyboard. As with the standard stop-signal task (see above), 75% of the trials were go trials (go stimuli were uninterrupted), whereas the remaining 25% of the trials were stop trials. The types of images that were displayed differed according to experimental group: Participants who were allocated to the alcohol cue exposure group were repeatedly exposed to eight different images, four images that depicted beer in a glass and four images that depicted beer in a bottle, whereas participants who were allocated to the control group were repeatedly exposed to eight different images, four that depicted water in a glass and four that depicted water in a bottle. For both groups, the number of trials, the proportion of stop trials and the SSD tracking procedure were identical to the baseline stop-signal task described above. There was one deviation from the procedure used for the baseline stop-signal task: After every 16 trials of the cued stop-signal task, participants were instructed to sniff the beverage before resuming the task (see the “Procedure” section).

### Procedure

Participants attended laboratories on the university campus between 12:00 p.m. and 7:00 p.m. They entered a neutral laboratory and provided a breath alcohol reading; no participants provided a breath sample that was positive for alcohol. They then completed a battery of questionnaires: a retrospective alcohol diary (14-day timeline follow-back (TLFB; Sobell and Sobell 1992), the Alcohol Use Disorders Identification Task (AUDIT; Babor et al. 2001), the Temptation and Restraint Inventory (TRI; Collins and Lapp 1992) and the Barratt Impulsivity Scales (BIS; Patton et al. 1995). Subjective alcohol craving was assessed with the ‘right now’ version of the Approach and Avoidance of Alcohol Questionnaire (AAAQ; McEvoy et al. 2004), which includes three subscales: inclined-indulgent (mild inclinations to drink; *α* = 0.87), obsessed-compelled (strong inclinations to drink; *α* = 0.83) and resolved-regulated (inclinations to avoid alcohol; *α* = 0.73). Subjective mood was assessed using the Brief Mood Introspection Scale (BMIS; Mayer and Gaschke 1988), which includes four subscales that reflect the affective dimensions of pleasant-unpleasant, negative-relaxed, arousal-calm and positive-tired. Participants then completed the baseline stop-signal task.

Following completion of the baseline stop-signal task, participants were randomly allocated to the alcohol cue exposure group or the control group. Participants in the control group were relocated to a second neutral laboratory where they completed the cued stop-signal task with embedded neutral (water) cues. After every 16 trials of the task, participants were signalled to raise a glass of water, sniff it and let the water touch their lips, but refrain from drinking. Participants in the alcohol cue exposure group were relocated to a different laboratory, a ‘bar lab’, to complete the cued stop-signal task with embedded alcohol-related cues. The bar lab is a purpose-built laboratory including beer pumps, alcohol advertisements and a variety of beverages on show (see https://www.liverpool.ac.uk/psychology-health-and-society/facilities/bar-lab/). After every 16 trials of the task, participants were signalled to raise a glass of beer, sniff it and allow it to touch their lips, but refrain from drinking. All participants complied with these instructions.

After participants completed the cued stop-signal task, they remained in the laboratory and completed the AAAQ and BMIS again. They were then asked to rate their level of thirst on a 100-mm visual analogue scale with anchors at 0 (not thirsty at all) to 100 (extremely thirsty) before completing an ad libitum alcohol taste test. The researcher presented 300 ml each of three different and distinctly flavoured types of beer (‘Becks’, 5% alcohol by volume (ABV); ‘Hoegaarden’, 4.9% ABV and ‘Old Golden Hen’, 4.1% ABV) in unmarked glasses. Participants were provided with visual analogue rating scales for each beer and instructed to rate each one on a series of adjectives (e.g. pleasant, fizzy). They were instructed to consume as little or as much beer as they liked in order to make accurate judgements and were given a maximum of 30 min to complete the rating scales. This procedure or slight variations thereof have been used to measure the motivation to consume alcohol in the laboratory and have good construct validity and sensitivity to experimental manipulations (Jones et al. 2016a, b). Before starting the taste test, participants were informed that after the taste test, they would complete a further cognitive task in which they could win small monetary rewards and that alcohol was known to have a detrimental effect on the performance of that task. This (false) information was provided in order to motivate participants to restrict their alcohol consumption during the taste test (see Christiansen et al. 2012a, b; Muraven et al. 2002), but participants were never actually required to complete an additional task. Following completion of the ad libitum taste test, participants were given a funnelled debrief, which asked multiple-choice questions about the purposes of the taste test, experimental manipulation and the stop-signal task (Jones et al. 2011a, 2011b); results are reported in the supplementary materials. Finally, participants were thanked, debriefed and received course credit or £10 in high-street shopping vouchers for their participation.

### Statistical analyses

#### Extraction of key variables from the baseline and cued stop-signal tasks

Inhibition error data suggests that participants understood the task instructions and were fully engaged with the task and that the dynamic tracking procedure was effective. On average, participants failed to inhibit on 48% of the trials during the baseline task and 46% of the trials during the cued task. Reaction times on go trials were subjected to a trimming procedure, similar to that applied in previous studies that used the stop-signal task (e.g. Verbruggen and De Houwer 2007): Trials with reaction times faster than 100 ms, slower than 2000 ms and more than three standard deviations above the mean for that task (baseline or cued) were removed prior to the calculation of the mean go reaction time. Stop-signal reaction time (SSRT) was calculated using the integration method (Verbruggen and Logan 2009), which involves subtracting the mean stop-signal delay (SSD) from the *N*th reaction time. *N* is calculated by ranking go RTs from the fastest to the slowest, then multiplying the total number of go trials (144 in both baseline and cued stop-signal tasks) by the proportion of stop trials on which that participant failed to inhibit. For example, if a participant failed to inhibit on 25% of the stop trials, the *N*th RT for this participant would be their 36th fastest go trial (144 × 0.25 = 36). SSRT would then be calculated by subtracting the mean SSD for that participant from this *N*th reaction time. Higher values of SSRT indicate worse inhibitory control. We computed the internal reliability (Cronbach’s *α*) of our tasks based on SSRT estimates on each of the three subblocks of each task. The cued versions of the task had good internal reliability (alcohol-cued, *α* = 0.82; neutral-cued, *α* = 0.86), whereas the reliability of the standard task (administered at baseline) was slightly below acceptable levels (*α* = 0.57).

#### Mediation analysis

We used PROCESS (Hayes 2012) to investigate if the influence of alcohol cue exposure on the amount of alcohol consumed during the taste test was mediated by inhibitory control and craving for alcohol. A composite craving measure was derived by averaging AAAQ inclined-indulgent and obsessed-compelled subscale scores because (1) the two were highly correlated (*r* = 0.71 *p* < 0.01), (2) alcohol cue exposure had robust effects on both of these subscales but not the resolved-regulated subscale and (3) some previous studies that used the AAAQ suggest that they may load onto the same factor (Klein et al. 2007). We calculated bias-corrected, bootstrapped (1000 samples) confidence intervals (Hayes 2012).

## Results

### Participant characteristics and dependent variables at baseline (Table 1)

Group differences in typical alcohol consumption, AUDIT scores, scores on the TRI and BIS, SSRT and go RTs at baseline were analysed using independent *t* tests with a conservative *α* = 0.01 to reduce the likelihood of a type 1 error. There were no significant group differences in any of these variables (*ts*(79) < 1.88, *ps* > 0.06), and gender ratios were comparable across groups (*χ* ^2^ = 0.15, *p* = 0.70).

**Table 1:** Participant characteristics and baseline variables

	Control (*N* = 44)	Alcohol cue exposure (*N* = 37)
Gender (female/male)	28/16	22/15
Age	20.23 (3.65)	19.73 (2.14)
Alcohol units/week	34.72 (16.78)	29.98 (21.10)
Heavy drinking days/week	2.31 (0.90)	2.09 (0.80)
Non-drinking days/week	3.85 (1.07)	4.07 (0.85)
AUDIT	15.14 (5.63)	13.05 (4.03)
BIS-non-planning	26.48 (5.25)	25.24 (5.24)
BIS-motor	25.25 (3.94)	23.78 (3.68)
BIS-attention	19.02 (2.98)	18.84 (2.37)
TRI-CEP	26.54 (11.32)	23.89 (11.57)
TRI-CBC	15.88 (6.78)	14.27 (6.33)
Baseline SSRT	183.27 (47.86)	194.91 (60.73)
Baseline Go RT	575.59 (142.17)	517.61 (133.77)

Values are means (standard deviations)

*Alcohol units/week* number of units of alcohol consumed as a weekly average, *Heavy drinking days* number of days per week in which participants consumed more than 6 units/48 g alcohol (females) or 8 units/64 g alcohol (males) (Office for National Statistics, 2015), *Non-drinking days/week* number of days per week in which participants abstained from alcohol, *AUDIT* Alcohol Use Disorders Identification Task, *BIS* Barratt Impulsivity Scale, *TRI* Temptation and Restraint Inventory, *CEP* Cognitive Emotional Preoccupation, *CBC* Cognitive Behavioural Control, *SSRT* stop-signal reaction time; *Go RT* reaction time on ‘go’ trials during the stop-signal task

### Craving and mood (Table 2)

AAAQ and BMIS data were missing for one participant in the control group. Changes in craving were assessed using a 3 (AAAQ subscales: inclined-indulgent, obsessed-compelled and resolved-regulated) × 2 (time: pre-manipulation and post-manipulation) × 2 (groups: alcohol cue exposure and control) mixed ANOVA. The main effects of time and subscale (*Fs* > 10.62, *ps* < 0.01), time and group (*F*(1, 78) = 25.51, *p* < 0.01, *η*_*p*_^*2*^ = 0.22) and subscale and time (*F*(2, 156) = 16.07, *p* < 0.01, *η*_*p*_^*2*^ = 0.17) interactions were all subsumed under a significant subscale × time × group interaction (*F*(2, 156) = 14.22, *p* < 0.01, *η*_*p*_^*2*^ = 0.15). To examine the interactions, we ran 2 (time: pre-manipulation and post-manipulation) × 2 (groups: alcohol cue exposure and control) mixed ANOVAs on each subscale individually. For both the *inclined-indulgent* and *obsessed*-*compelled* subscales, there was a significant time × group interaction (*Fs* > 26.03, *ps* < 0.01). The nature of the interaction was consistent across both subscales with scores increasing in the alcohol cue exposure group (*inclined-indulgent* (*t*(36) = 5.21, *p* < 0.01, *d* = 0.86); *obsessed-compelled* (*t*(36) = 4.65, *p* < 0.01, *d* = 0.77)), but not the control group (*ts* < 1.47, *ps* > 0.15). For the *resolved-regulated* subscale, there was a main effect of time (*F*(1, 78) = 4.38, *p* = 0.04, *η*_*p*_^*2*^ = 0.05) with scores reducing from pre-manipulation to post-manipulation in both groups (*t*(79) = 2.10, *p* = 0.04, *d* = 0.24), but no significant time × group interaction (*F*(1, 78) < 0.01, *p* = 0.99). These findings indicate that, overall, our manipulation was successful because exposure to a combination of visual, olfactory and contextual alcohol-related cues led to increased craving for alcohol.

**Table 2:** Scores on AAAQ and BMIS questionnaires both pre-manipulation and post-manipulation, split by experimental group

	Control		Alcohol cue exposure	
	Pre-manipulation	Post-manipulation	Pre-manipulation	Post-manipulation
AAAQ: Inc	4.86 (2.00)	4.66 (2.06)	4.29 (1.41)	5.24 (1.66)
AAAQ: Obs	1.27 (1.44)	1.26 (1.56)	0.97 (1.06)	1.99 (1.64)
AAAQ: Res	1.08 (0.88)	0.95 (0.99)	1.04 (0.97)	0.91 (0.83)
BMIS-pleasant	5.33 (6.84)	4.56 (6.10)	6.89 (5.50)	7.16 (5.00)
BMIS-negative	6.63 (2.86)	6.72 (2.51)	6.00 (2.89)	5.57 (2.46)
BMIS-positive	7.72 (3.74)	7.09 (3.15)	8.35 (2.70)	8.24 (2.34)
BMIS-arousal	16.79 (3.23)	16.44 (2.61)	16.49 (3.12)	15.97 (2.55)

Values are means (standard deviations)

*AAAQ* Approach and Avoidance of Alcohol Questionnaire, *Inc* inclined-indulgent subscale, *Obs* obsessed-compelled subscale, *Res* resolved-regulated subscale, *BMIS* Brief Mood Introspection Scale

Changes in mood were assessed using a 4 (BMIS subscales: pleasant-unpleasant, negative-relaxed, positive-tired and arousal-calm) × 2 (time: pre-manipulation and post-manipulation) × 2 (groups: alcohol cue exposure and control) mixed ANOVA. There was a significant main effect of the subscale (*F*(3, 234) = 159.83, *p* < 0.01, *η*_*p*_^*2*^ = 0.67) with scores on the arousal-calm subscale higher than all others (*ps* < 0.01); however, there were no interactions involving time or group (*Fs* < 2.37, *ps* > 0.13). Therefore, our cue exposure manipulation did not influence self-reported mood.

### Inhibitory control (Fig. 1)

Changes in SSRT were analysed using a 2 (tasks: baseline and cued) × 2 (groups: alcohol cue exposure and control) mixed ANOVA. There was a significant main effect of the task (*F*(1, 79) = 39.48, *p* < 0.01, *η*_*p*_^*2*^ = 0.33), as SSRT was slower (indicating impaired inhibitory control) in the cued compared to the baseline task in both the alcohol cue exposure (*t*(36) = 4.43, *p* < 0.01, *d* = 0.73) and control (*t*(43) = 4.59, *p* < 0.01, *d* = 0.69) groups. More importantly, this main effect was qualified by a significant task × group interaction (*F*(1, 79) = 4.54, *p* = 0.04, *η*_*p*_^*2*^ = 0.05) which arose because at baseline, groups did not differ in SSRT (*t*(79) = 0.96, *p* = 0.34, *d* = 0.15), whereas at post-manipulation, there was a significant difference (*t*(79) = 3.29, *p* < 0.01, *d* = 0.51) with the alcohol cue exposure group having slower SSRT indicative of impaired inhibitory control.

**Fig. 1:**
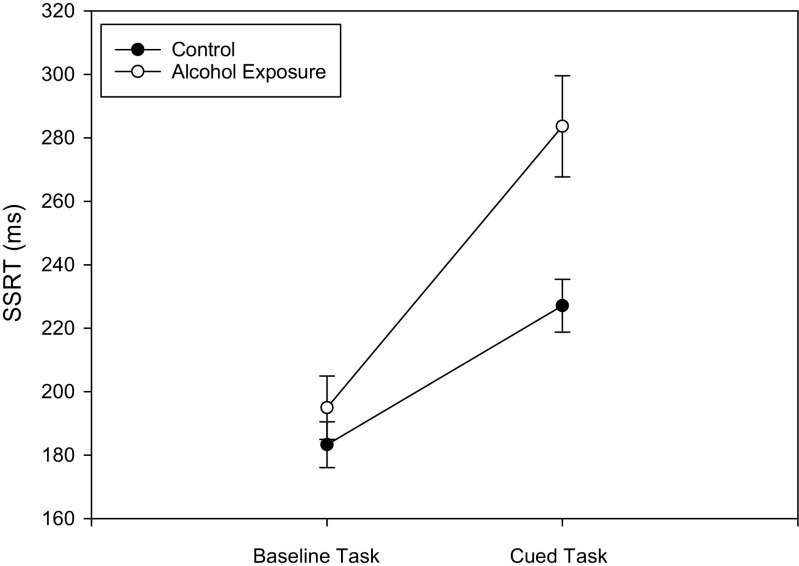
Stop-signal reaction time in the baseline and the cued stop-signal tasks. Values are means; *bars* represent standard errors

Changes in mean reaction time on go trials were analysed using a 2 (tasks: baseline and cued) × 2 (groups: alcohol cue exposure and control) mixed ANOVA. There was a significant main effect of the task (*F*(1, 79) = 140.92, *p* < 0.001, *η*_*p*_^*2*^ = 0.64): Overall, go reaction times were slower during the cued task (700.45 ± 105.83 ms) compared to the baseline task (549.11 ± 140.57 ms; *t*(80) = 11.44, *p* < 0.01, *d* = 1.21). The main effect was qualified by a task × group interaction (*F*(1, 79) = 4.96, *p* = 0.029, *η*_*p*_^*2*^ = 0.06). The alcohol cue exposure and control groups did not differ on go reaction time during either the baseline task (*t*(79) = 1.87, *p* = 0.06) or the cued task (*t*(79) = 0.11, *p* = 0.99), and both groups were significantly slower during the cued task compared to the baseline task (alcohol cue exposure group, *t*(36) = 7.72, *p* < 0.01, *d* = 1.27; control group, *t*(43) = 9.61, *p* < 0.01, *d* = 1.43). The interaction arose because the magnitude of the latter effect (slowing of go reaction times during the cued task compared to the baseline task) was larger in the alcohol cue exposure group compared to the control group (182.69 ± 144.02 vs. 129.97 ± 86.25 ms; *t*(79) = 2.22, *p* = 0.03, *d* = 0.34).

### Ad libitum alcohol consumption

Levels of thirst did not significantly differ across groups (control group 60.25 ± 21.43 ml, alcohol cue exposure group 58.24 ± 18.82 ml; *t*(79) = 0.44, *p* = 0.66). The alcohol cue exposure group consumed more alcohol than the control group on average, although this group difference was not statistically significant (236.49 ± 123.54 vs. 194.25 ± 150.12 ml; *t*(79) = 1.37, *p* = 0.09, 95% CI = 103.79–19.32, *d* = 0.38).

### Gender differences

Note that our study was not powered to detect group × gender interactions. Aside from a significant group difference in ad libitum alcohol consumption (men drank more than women) and a task × group × gender interaction for go reaction time, there were no other main effects of gender, or gender × group interactions, for any of our outcome variables. See the supplementary materials for descriptive and inferential statistics.

### Mediation analyses (Fig. 2)

The direct effect of alcohol cue exposure on absolute alcohol consumption was not significant (95% CI = 0.10 to 0.14), confirming the findings from the *t* test reported above. However, the indirect effects of both SSRT (95% CI = 0.01 to 0.12) and craving (95% CI = 0.01 to 0.10) were statistically significant. This analysis demonstrates that although alcohol cue exposure did not lead to a robust increase in alcohol consumption compared to the control manipulation, there was an indirect effect: Participants who demonstrated reduced inhibitory control and increased craving following alcohol cue exposure consumed more alcohol overall. Finally, we also repeated this analysis with the addition of go reaction times as an additional mediator. This variable did not have a significant indirect effect on alcohol consumption (95% CI = 0.05 to 0.08), yet the indirect effects of both SSRT and craving remained statistically significant.

**Fig. 2:**
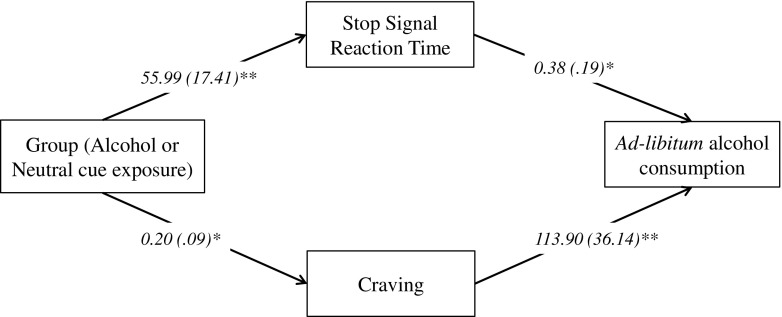
A multiple-mediation pathway of the indirect effect of the experimental group on ad libitum alcohol consumption via inhibitory control and craving. Values are regression coefficients and standard errors. *p < 0.05; **p < 0.01

## Discussion

In this study, we demonstrated that participants who were exposed to alcohol-related cues had impaired inhibitory control and elevated subjective craving compared to a control group of participants who were exposed to water-related (control) cues. The group which was exposed to alcohol cues also consumed more alcohol during a bogus taste test compared to the control group although this difference was not statistically significant. Most importantly, although alcohol cue exposure did not lead to a robust increase in alcohol consumption, alcohol cue exposure had an indirect effect: Participants who demonstrated reduced inhibitory control and reported elevated craving following alcohol cue exposure consumed more alcohol overall.

Our findings provide clear support for our first hypothesis that exposure to alcohol-related cues would provoke an impairment in inhibitory control. This builds on a number of previous demonstrations that exposure to alcohol-related cues presented in a range of modalities (visual, contextual and olfactory) results in impairments in inhibitory control (Czapla et al. 2015; Gauggel et al. 2010; Jones and Field 2015; Monk et al. 2016; Petit et al. 2012; Weafer and Fillmore 2012a, 2014; Zack et al. 2011), although this effect may be very transient and may not be detectable if inhibitory control is measured after (rather than during) exposure to alcohol cues (Jones et al. 2013b). The effect of alcohol cues on inhibitory control may be a more generalised phenomenon, extending beyond addiction, because appetitive cues in general may interfere with the ability to exercise inhibitory control (Guitart-Masip et al. 2014). We also demonstrated impaired inhibitory control (albeit of a reduced magnitude) in a control group of participants who were exposed to water-related cues, relative to the performance of the same participants during a standard stop-signal task (that did not contain any cues). This observed impairment of inhibitory control might be attributed to the increased complexity of the cued task compared to the baseline task, fatigue (Persson et al. 2007) or self-control depletion (Hagger et al. 2010) caused by the completion of the stop-signal task at baseline.

Contrary to our hypotheses, participants who were exposed to alcohol-related cues did not consume more alcohol during the bogus taste test compared to the control group of participants who were exposed to water-related cues. This finding is in contrast to our previous study (Jones et al. 2013a, b) and other laboratory (MacKillop and Lisman 2007) and naturalistic investigations (Koordeman et al. 2011) which demonstrated that alcohol cue exposure increases alcohol consumption (see Veilleux and Skinner 2015, for a review). However, the inconsistency between the present findings and previous findings may be attributable to a feature of the methodology used in the present study, because we deliberately motivated participants to attempt to restrict their alcohol consumption during the taste test (by making them believe that drinking too much could limit the amount of money they could earn on a subsequent cognitive task, cf. Muraven et al. (2002)). An unintended consequence of this manipulation is that it may have imposed a ceiling effect on alcohol consumption in the sample as a whole. In support of this explanation, participants in this study consumed, on average, only 214 ml of the 900 ml of beer that was available to them (24%), which is lower than in many of the previous studies (e.g. Jones et al. 2013a, 2013b; MacKillop and Lisman 2007). We deliberately prompted participants to attempt to restrict their alcohol consumption because, in principle, if participants are not motivated to limit how much they drink, then this could obscure associations between inhibitory control and alcohol consumption (Hofmann et al. 2012).

In support of our third hypothesis, we demonstrated that the change in inhibitory control (from the baseline task to the cued stop-signal task) partially mediated the effect of alcohol cue exposure on subsequent alcohol consumption. To our knowledge, this is the first study to demonstrate this effect, which provides empirical support for claims that transient impairments (‘state fluctuations’) in inhibitory control during ‘high-risk’ situations (such as during exposure to alcohol-related cues) will increase the likelihood of drinking behaviour or increase the amount of alcohol consumed (de Wit 2009; Jones et al. 2013a, b). In addition to its theoretical importance, a practical implication of this demonstration is that it suggests that behavioural interventions (such as inhibitory control training (ICT)) which attempt to strengthen associations between alcohol-related cues and the engagement of inhibitory control (through repeated pairing of the two) might be a useful intervention for alcohol use disorders and other addictions (see Allom et al. (2015) and Jones et al. (2016a, b) for meta-analyses of proof-of-concept demonstrations of this intervention in the laboratory). Alternatively, screening of alcohol-dependent patients for their sensitivity to the disinhibiting effects of alcohol cues may help to identify those who would benefit from cognitive behavioural therapy techniques that aim to identify and plan coping responses for high-risk situations (Ryan 2013). We also demonstrated that changes in alcohol craving (alongside changes in inhibitory control) also partially mediated the effects of alcohol cue exposure on alcohol consumption during the taste test, which is consistent with previous EMA studies (Fatseas et al. 2015; Serre et al. 2015).

Future research should investigate the mechanism(s) through which alcohol-related cues impair inhibitory control. There are a number of plausible explanations, which are not mutually exclusive. For example, involuntary attentional capture by alcohol-related cues may occupy cognitive resources that are essential for the engagement of inhibitory control (Pessoa et al. 2012; Weafer and Fillmore 2012b), or inhibitory control resources may be ‘depleted’ if they are engaged to resist subjective craving provoked by substance-related cues (Gauggel et al. 2010; Muraven and Shmueli 2006). We note that the resource model of self-control is currently disputed. However, alternative conceptualisations, such as the notion that *perceptions* of self-control effort determine the motivation, rather than the ability, to exercise self-control in the future (Christiansen et al. 2012a; Inzlicht et al. 2014; Lurquin et al. 2016), could also account for the interrelationships between alcohol cue exposure, inhibitory control and alcohol consumption.

Limitations of the current study include our decision to combine different cue exposure modalities (olfactory, visual and contextual) to investigate their influence on inhibitory control and alcohol consumption. Whilst this is likely to be representative of the way in which alcohol-related cues are encountered outside of the laboratory, it does mean that we are unable to distinguish the disinhibiting effects of alcohol cues presented in these different modalities (cf. Monk et al. 2016). Future studies could use crossover designs to examine the effects of specific cues in isolation and in combination. Second, we did not measure participants’ smoking behaviour so we could not consider the influence of smoking in our analyses. Given that cigarette smoking is associated with impaired inhibitory control (Luijten et al. 2011), future studies of this type should attempt to statistically control for this influence. Finally, our participants were all heavy drinkers, and we did not include control groups of light drinkers or abstainers in order to investigate if the effects reported here are seen in all alcohol consumers or are limited to heavy drinkers only. Previous studies that investigated the effects of alcohol cues on inhibitory control in heavy vs. light drinkers have reported conflicting findings (Czapla et al. 2015; Jones and Field 2015; Kreusch et al. 2013; Nederkoorn et al. 2009), so it is important to clarify this issue in future research.

To conclude, we demonstrated that exposure to alcohol-related cues prompts transient impairments in inhibitory control and increases in subjective craving, and these effects indirectly mediate the effects of alcohol cues on subsequent voluntary alcohol consumption. These findings are the very first demonstration that transient fluctuations in inhibitory control are one mechanism through which substance-related cues can increase substance use behaviour. Further work is required to establish the mechanism and generality of these effects and to exploit the practical applications of this work in the search for new behavioural interventions for alcohol use disorders and other addictions.

## Electronic supplementary material

ESM 1 (DOCX 28 kb)
