# Supplementary material for: Elevated alcohol consumption following alcohol cue exposure is partially mediated by reduced inhibitory control and increased craving
Source: Psychopharmacology (Berl). 2017 Jul 25;234(19):2979–88. doi: 10.1007/s00213-017-4694-6 (PMC5591800; doi:10.1007/s00213-017-4694-6)
Supplement: Supplementary file 1 — (DOCX 28 kb) [file 213_2017_4694_MOESM1_ESM.docx]

**Elevated alcohol consumption following alcohol cue exposure is partially mediated by reduced inhibitory control and increased craving**

***Supplementary materials***

**Funnelled debriefing**

Following completion of the taste test, participants completed a funnelled debriefing questionnaire. This included an open ended question ‘*What was the purpose of this experiment?*’ in which participants were invited to write as much as they wanted. Following this, participants were shown 3 statements ‘*The computer tasks were designed to…*’, *The purpose of the drinks sniffing was to…*’, and ‘ *The purpose of the taste-test was to…*’. Each statement had 5 or 6 choices and participants were asked to select the correct statement. One statement was correct e.g. ‘*The purpose of the taste test was to…. measure how much alcohol I drank in response to the cue*s’, whilst the others were incorrect but not obviously so e.g. ‘*The purpose of the taste test was to… measure my preference for each drink*’.

We investigated whether awareness of the study overall, the purpose of the experimental manipulation or purpose of the taste test had a direct or moderating effect on any of our outcome measures. None of the participants in the alcohol cue exposure group demonstrated awareness of the overall aims of the study (inferred from their responses to the open ended question). Four participants in the cue exposure group were aware that the drink sniffing was designed to increase their craving for alcohol. Removal of these participants did not significantly influence the craving results reported in the manuscript: the subscale x time x group interaction remained significant (*F*(2, 156) = 14.21, *p* <.001, *η_p_^2^* = .15). Four participants (2 from the cue exposure group) correctly answered that the purpose of the computer task was to *‘Assess my behavioural impulsivity (response inhibition)’*, removal of these participants did not significantly influence the inhibitory control results reported in the manuscript: the group x time interaction (on SSRT) remained significant (F(1, 74) = 4.52, p = .038, *η_p_^2^ = .057.* Twenty-one participants (10 from alcohol cue exposure group, and 11 from the control group) reported awareness of the purpose of the taste test, i.e. they were aware that their alcohol consumption would be recorded, despite the cover story which claimed that it would not. We repeated the analysis of beer consumption during the taste test with an added between-subjects factor of awareness (aware, unaware), and this did not alter our results: the main effect of group remained non-significant, (*F*(1, 75) = 1.68, *p* = .198), and the group x awareness interaction was not significant (*F*(1, 75) < 0.01, *p* = .94).

**Gender differences**

*Participant characteristics at baseline (Supplementary Table 1)*

Each variable was analysed using a 2 x 2 ANOVA with between-subjects factors of gender and group, using a conservative α = .01 to reduce the likelihood of type 1 error. There were no significant main effects of gender (Fs < 2.43, ps > .12) or group x gender interactions (Fs < 3.73, ps > .06).

*Craving and mood (Supplementary table 2)*

The ANOVAs on AAAQ subscales were repeated with an additional between- subjects factor of gender. Notably, there was no significant main effect (F(1, 76) = 0.64, p = .42) and no significant interactions involving gender (Fs < 2.45, ps >.09). The ANOVAs on BMIS subscales were also repeated with an additional between-subjects factor of gender. There was no significant main effect of gender (F(1, 79) = 1.15, p = .29), however, there was a significant interaction between subscale x group x gender (F(3,228) = 3.27, p = .02, *η_p_^2^* = .04), but no significant subscale x time x gender x group interaction (F(3, 228) = 1.08, p = .36). Analysing the data separately for each group demonstrated a significant subscale x gender interaction in the control (F(3, 123) = 6.52, p < .01, *η_p_^2^* = .14) but not the alcohol cue exposure group (F(3,105) = 0.02, p = .99). After collapsing the subscales across time, we found that females in the control group had significantly higher scores on the pleasant subscale compared to males males (9.37 ± 9.46 vs. 3.20 ± 8.25; t(41) = 2.22, p = .03), and significantly lower scores on the negative subscale compared to males (8.77 ± 3.76 vs. 12.27 ± 3.22; t(41) = 3.50, p < .01). There were no gender differences on the other two subscales (ts < 1.61, ps > .11).

*Inhibitory control (Supplementary table 2)*

The ANOVAs on SSRT and Go reaction time were repeated with an additional between-subjects factor of gender. Most importantly, there was no significant main effect of gender (F(1,77) = 0.59, p = .45), and no significant interactions involving gender for SSRT (Fs <0.97, ps >.33). For Go Reaction time there was a significant task x group x gender interaction (F(1,77) = 11.16, p = .01, *η_p_^2^* = .13). In males the task x group interaction was significant (F(1, 29) = 15.54, p < .01, *η_p_^2^* = .35), but this interaction was not significant in females (F(1, 48) = 0.70, p = .79). Among male participants, at baseline, Go reaction times Go reaction times were significantly faster in the alcohol cue exposure group compared to the control group (t(29) = 3.12, p < .01, *d* = 1.12). However, Go reaction times among male participants during the cued task did not differ between groups (t(29) = 0.98, p = .33, *d* = 0.34). Furthermore, among males, Go reaction times were also significantly slower in the cued task in both groups (control: t(14) = 7.48, p < .01, *d* = 1.08; alcohol-cue exposure: t(15) = 4.32, p < .01, *d* = 1.93 ).

*Ad-libitum alcohol consumption (Supplementary table 2)*

The ANOVA on alcohol consumption was repeated with an additional between-subjects factor of gender. There was a significant main effect of gender (F(1, 77) = 12.71, p = .001, *η_p_^2^* = .14). Men drank significantly more alcohol than women (280.22 ± 23.34ml vs. 174.04 ± 18.50ml; d = 0.82). Importantly, the gender x group interaction was not statistically significant (F(1,77) = 0.65, p = .42, *η_p_^2^* = .01).

Supplementary Table 1: Participant characteristics and baseline variables, split by gender. Values are means (Standard Deviations).

**Control (N =44) Alcohol cue exposure (N = 37)**

**Males Females Males Females**

Age 20.06 (3.57) 20.29 (3.77) 20.27 (2.58) 19.36 (1.76)

Alcohol units / week 68.19 (23.43) 70.14 (38.60) 66.47 (14.31) 55.50 (24.00)

Heavy Drinking Days / week 1.94 (0.66) 2.54 (0.96) 2.03 (0.61) 2.14 (0.92)

Non Drinking Days / week 3.91 (1.36) 3.82 (0.89) 4.33 (0.90) 3.89 (0.79)

AUDIT 14.75 (6.02) 15.36 (10.32) 13.27 (2.74) 12.91 (4.77)

BIS – Non Planning 27.19 (5.33) 26.07 (5.26) 26.80 (4.59) 24.18 (5.48)

BIS – Motor 25.38 (3.86) 25.18 (4.05) 24.07 (3.08) 23.59 (4.09)

BIS – Attention 19.06 (3.64) 19.00 (2.59) 18.47 (2.10) 19.09 (2.56)

TRI – CEP 27.94 (13.14) 25.75 (10.32) 19.27 (7.95) 27.05 (12.71)

TRI – CBC 17.56 (8.21) 14.93 (5.75) 13.33 (6.11) 14.91 (6.53)

*Alcohol units / week = Number of units of alcohol consumed as a weekly average; Heavy Drinking Days = number of days per week in which participants consumed more than 6 units / 48g alcohol (females) or 8 units / 64g alcohol (males) (Office for National Statistics, 2015); Non Drinking Days / week = number of days per week in which participants abstained from alcohol; AUDIT = Alcohol Use Disorders Identification Task; BIS = Barratt Impulsivity Scale; TRI = Temptation and Restraint Inventory (CEP = Cognitive Emotional Preoccupation; CBC = Cognitive Behavioural Control);*

Supplementary Table 2: Outcome measures, split by gender and group. Values are means (Standard Deviations).

**Control Alcohol cue exposure**

**Pre-manipulation Post-manipulation Pre-manipulation Post-manipulation**

**Males Females Males Females Males Females Males Females**

AAAQ: Inc 4.53 (2.04) 5.04 (2.00) 3.93 (2.26) 5.04 (1.87) 4.37 (1.47) 4.24 (1.40) 5.41 (1.74) 5.12 (1.63)

AAAQ: Obs 1.07 (1.16) 1.38 (1.57) 1.03 (1.72) 1.38 (1.74) 0.78 (0.88) 1.10 (1.17) 1.80 (1.52) 2.13 (1.74)

AAAQ: Res 1.17 (1.09) 1.03 (0.76) 1.11 (1.28) 0.86 (0.81) 1.01 (1.11) 1.05 (0.88) 0.77 (0.85) 1.00 (0.82)

BMIS-Pleasant 1.86 (5.82) 7.17 (6.71) 2.27 (7.03) 5.79 (5.27) 6.60 (5.39) 7.09 (5.68) 7.07 (4.99) 7.23 (5.12)

BMIS-Negative 8.40 (2.16) 5.68 (2.76) 7.73 (2.66) 6.18 (2.29) 5.87 (3.20) 6.09 (2.72) 5.53 (2.23) 5.59 (2.65)

BMIS-Positive 6.40 (3.20) 8.43 (3.87) 6.20 (3.41) 7.57 (2.96) 8.33 (2.55) 8.36 (2.85) 8.27 (2.28) 8.23 (2.43)

BMIS-Arousal 17.40 (2.97) 16.46 (3.36) 16.80 (2.21) 16.25 (2.82) 16.26 (3.26) 16.63 (3.09) 16.00 (1.96) 15.95 (2.94)

SSRT 176.35 (37.95) 187.23 (52.95) 233.48 (63.39) 233.43 (51.33) 211.63 (62.19) 183.51 (58.37) 287.24 (69.02) 281.25 (113.64)

Go RT 582.51 (127.37) 571.63 (152.09) 679.01 (99.81) 712.88 (112.80) 462.22 (80.77) 555.38 (150.47) 717.52 (117.78) 688.58 (95.05)

Alcohol cons (ml) - - 246.56 (188.64) 164.36 (116.50) - - 313.87 (134.09) 183.73 (83.59)

*AAAQ = Approach and Avoidance of Alcohol Questionnaire (Inc = inclined-indulgent subscale, Obs = obsessed-compelled subscale; Res = resolved-regulated subscale); Alcohol cons = millilitres of alcohol consumed during the ad-libitum taste test; BMIS = Brief Mood Introspection Scale; SSRT = Stop Signal Reaction time; Go RT = reaction time on ‘Go’ trials during the Stop Signal task; Alcohol cons. = volume of alcohol consumed during the bogus taste test, in millilitres.*
